# Highly Sensitive Surface Plasmon Resonance Humidity Sensor Based on a Polyvinyl-Alcohol-Coated Polymer Optical Fiber

**DOI:** 10.3390/bios11110461

**Published:** 2021-11-17

**Authors:** Ying Wang, Jingru Wang, Yu Shao, Changrui Liao, Yiping Wang

**Affiliations:** 1 Key Laboratory of Optoelectronic Devices and Systems of Ministry of Education, College of Physics and Optoelectronic Engineering, Shenzhen University, Shenzhen 518060, China; 2014170126@email.szu.edu.cn (J.W.); shaoyu2016@email.szu.edu.cn (Y.S.); cliao@szu.edu.cn (C.L.); ypwang@szu.edu.cn (Y.W.); 2 Shenzhen Key Laboratory of Photonic Devices and Sensing Systems for Internet of Things, Guangdong and Hong Kong Joint Research Centre for Optical Fibre Sensors, Shenzhen University, Shenzhen 518060, China

**Keywords:** optical fiber sensor, surface plasmon resonance, humidity sensing, breath monitoring

## Abstract

A surface-plasmon-resonance-based fiber device is proposed for highly sensitive relative humidity (RH) sensing and human breath monitoring. The device is fabricated by using a polyvinyl alcohol (PVA) film and gold coating on the flat surface of a side-polished polymer optical fiber. The thickness and refractive index of the PVA coating are sensitive to environmental humidity, and thus the resonant wavelength of the proposed device exhibits a redshift as the RH increases. Experimental results demonstrate an average sensitivity of 4.98 nm/RH% across an ambient RH ranging from 40% to 90%. In particular, the sensor exhibits a linear response between 75% and 90% RH, with a sensitivity of 10.15 nm/RH%. The device is suitable for human breath tests and shows an average wavelength shift of up to 228.20 nm, which is 10 times larger than that of a silica-fiber-based humidity sensor. The corresponding response and recovery times are determined to be 0.44 s and 0.86 s, respectively. The proposed sensor has significant potential for a variety of practical applications, such as intensive care and human health analysis.

## 1. Introduction

Monitoring the relative humidity (RH) of the environment can be critical in a variety of fields, such as agriculture, food production, medical research, weather forecasting, and air quality analysis [1]. The design and fabrication of high-sensitivity RH sensors have attracted increased attention in recent years. Optical fiber sensors provide several advantages over conventional electrical sensors, such as integrated miniaturization and good resistance to electromagnetic interference [2]. Various optical fiber structures have been used recently in the design of RH sensors, including interferometers [3,4,5,6,7,8], long-period fiber gratings [9,10], fiber Bragg gratings [11,12,13,14], microfibers [15,16,17,18,19], and polymer fibers [20,21,22,23]. These structures can be broadly divided into two types: intensity modulated and wavelength modulated. Intensity-modulated devices are widely used in the design of RH sensors because they are simple and cost effective. For example, Miao et al. reported an RH sensor produced by coating a tilted fiber Bragg grating with a polyvinyl alcohol (PVA) film, achieving a sensitivity of 2.52 and 14.95 dBm/RH% for ambient RH ranges of 20–74% and 74–98%, respectively [24]. However, it is difficult to maintain stability and accuracy with intensity-modulated devices, due to factors such as system noise and light source fluctuations. Therefore, wavelength demodulation technology has been used to overcome these challenges.

PVA has been widely used in the design of wavelength-demodulated RH sensors. It can be easily dissolved in hot water and evenly forms a film after drying. As such, dip-coating processes are often used to coat fiber surfaces with PVA. The refractive index (RI) and volume of the PVA film change dramatically as it absorbs water from the surrounding environment. Wong et al. demonstrated a sensor fabricated by coating a photonic crystal fiber (PCF) with a PVA film, forming a Michelson interferometer. The sensitivity reached 0.3 nm/RH% for the RH ranging from 30% to 90% [25]. Tang et al. proposed a highly sensitive humidity sensor consisting of side-polished fibers coated with polymer nanostructures [26]. This design achieved a humidity sensitivity of up to 1.12 nm/RH%, which is the highest sensitivity reported to date. The designs mentioned above are generally fragile in structure due to the silica fibers they use. There is still much room for sensitivity improvement of the proposed device.

In this paper, we propose an RH sensor based on a PVA-coated, side-polished polymer optical fiber (POF) whose resonant wavelength exhibits a rapid redshift as the RH increases. The sensor is fabricated by coating the gold surface of a side-polished POF surface plasmon resonance (SPR) device with a thin PVA film using the dip-coating process. The resonant wavelength of the sensor can be determined by the thickness and RI of the PVA film in various environments. The sensitivity of the sensor reaches 4.98 nm/RH% as the RH varies from 40% to 90%. Additionally, the wavelength shift shows good linearity from 75% to 90% RH, with a sensitivity of 10.15 nm/RH%, which is significantly higher than previously reported values. This device exhibits several qualities that are beneficial for humidity-sensing applications, including high sensitivity, a simple structure, and fast response.

## 2. Device Fabrication

**Figure 1:**
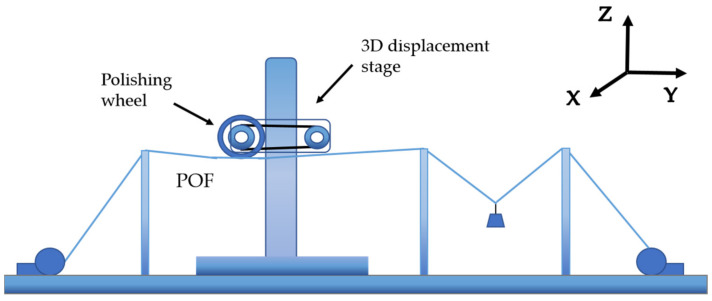
Schematic diagram of the optical-fiber-polishing system.

The SPR sensor was fabricated using a low-index POF (GigaPOF-62SR, Chromis Fiberoptics Inc., Chromis Technologies, NJ, USA). The fiber consisted of three layers, including a core, cladding, and over-cladding, with diameters of 62.5, 102.5, and 490 μm, respectively. The RI of the core/cladding was 1.356/1.342 nominated at 589 nm [27,28]. The POF could be polished to a D-shaped geometry using an optical-fiber-polishing system developed by our group [29]. Figure 1 shows the schematic diagram of the side-polishing equipment used here. The polishing wheel was placed on a three-dimensional (3D) displacement stage, which was able to move along the X, Y, and Z directions and was controlled by a computer. Several fiber holders were used to fix the POF and 20 g weights were used for a counterweight. During the polishing process, the polishing wheel reciprocated along the Y direction with a speed of 1 mm/s and a range of motion of 5 mm, and 1500, 5000, and 10,000-mesh abrasive paper was sequentially used to polish the POF for 15, 60, and 180 s, respectively. Note that these polishing parameters were optimized to achieve the expected polishing depth and flatness, considering time saving. Finally, the fabricated D-shaped POF exhibited a side-polished segment with a length of ~5 mm and a residual thickness of ~250 μm.

**Figure 2:**
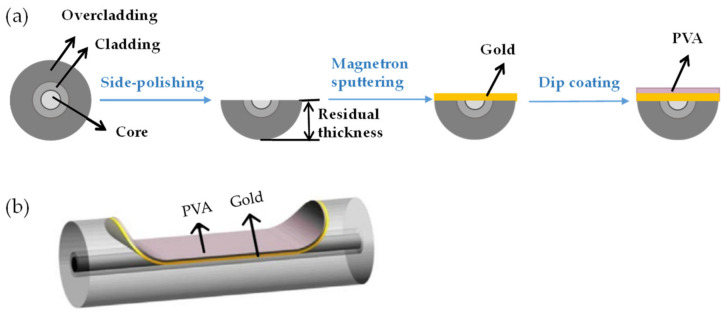
(**a**) Fabrication processes of the PVA-coated RH sensor in cross-sectional views. (**b**) 3D schematic view of the proposed sensor.

**Figure 3:**
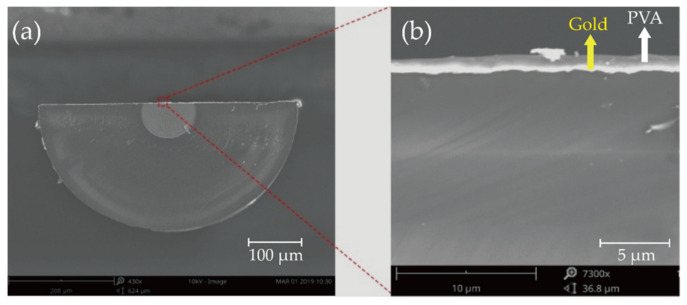
(**a**) SEM image of the PVA-coated RH sensor in cross-sectional view. (**b**) Enlarged view of the red area marked in (**a**).

The fabrication processes of the PVA-coated RH sensor are summarized in Figure 2a. The POF was first polished into a D-shaped geometry with a residual thickness of ~250 μm. The abovementioned “residual thickness”, as labeled in Figure 2a, is used here to describe the cross-sectional size of the D-shaped region after polishing. A 50-nm-thick gold film was then deposited onto the flat surface of the D-shaped segment using the magnetron sputtering technique. The fabricated SPR sensor was then cleaned and immersed in a prepared PVA solution and coated with a PVA film through the single dip-coating technique with a constant withdrawal speed of 4 cm/s [30]. The aqueous PVA solution with weight-to-weight concentrations ranging from 0.5% to 2% (wt/wt) was prepared by dissolving PVA particles in deionized water, with a magnetic agitator stirring the solution for 1 h at 90 °C. After the single dip-coating, a homogeneous PVA film was formed on the sensor surface after drying at 80 °C for 2 h. Figure 2b shows the 3D schematic view of the device obtained after the processes shown in Figure 2a. Furthermore, a sample fabricated with a PVA concentration of 2% (wt/wt) was cleaved at the D-shaped region and observed by scanning electron microscopy (SEM, FEI Scios); the coated layer on the fiber flat surface can be seen from the SEM image, as shown in Figure 3. Note that no apparent interface between the gold film and the PVA film can be observed from the image shown in Figure 3b, because the gold film was too bright during imaging. During the RH test, the sensor was placed in a homemade environmental chamber at room temperature. One end of the sensor was connected to a halogen light source (400–2000 nm, Ocean Optics, Ocean Insight, Shanghai, China), and the other end was connected to an optical fiber spectrometer (NIRQuest512, Ocean Spectra, Ocean Insight, Shanghai, China). The homemade chamber contained a humidifier, a drier, a fan, and an electric hygrometer (HP22-A, Rotronic Instrument Corp., Rotronic AG, Bassersdorf, Switzerland). These devices were used to control the RH between 40% and 90%, with an accuracy of 0.1%. RH data were collected by the electric hygrometer, and transmission spectra were measured by the spectrometer. All collected data were recorded in real time on a personal computer (PC) during the tests.

Three samples with a polishing length of 5 mm, a residual thickness of 250 μm, and a gold film thickness of 50 nm were prepared to investigate the sensing performance of the device for varying PVA film thicknesses, produced using PVA solutions of 0.5%, 1%, and 2% (wt/wt), respectively, as listed in Table 1. The initial resonant wavelengths of these samples were 613, 712, and 1380 nm, respectively, in indoor air with an RH of 55%. 

## 3. Results and Discussion

**Figure 4:**
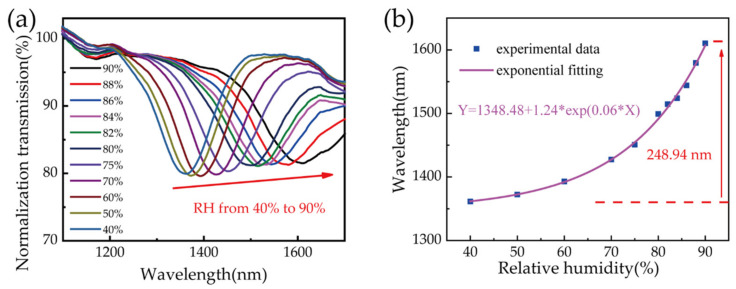
(**a**) Evolution of the transmission spectrum with increasing humidity for sample 3. (**b**) Resonance wavelengths of sample 3 for varying RH values. The purple and blue curves in (**b**) represent exponential and linear fits applied to the experimental data, respectively.

A resonant dip was observed in the device transmission spectrum after PVA coating, which shifted as the ambient RH varied. Sample 3 was prepared with a 2% (wt/wt) PVA solution, and the transmission spectrum showed a resonant dip at 1379.94 mm after the curing process. This dip shifted to longer wavelengths as the ambient RH in the chamber increased. Figure 4a shows the evolution of the transmission spectrum for RH values changing from 40% to 90%. Theoretically, the RI of the PVA coating decreases with an increase in the ambient RH, inducing a blueshift of the resonant wavelength [31]. However, the PVA coating on the fiber surface is thin, and it swells significantly due to moisture absorption [32]. This resulted in a thicker film and a redshift in the resonant wavelength. 

Figure 4b shows the relationship between resonant wavelength and ambient RH. The solid black squares are resonant wavelengths corresponding to each humidity level. As the RH varied from 40% to 90%, the resonant dip shifted from 1361.37 to 1610.31 nm. The total wavelength shift was 248.94 nm, and the averaged sensitivity was as high as 4.98 nm/RH%. The experimental data could be fitted well with an exponential function, as the pink curve shown in Figure 4b. However, the resonant wavelength shift was approximately linear in response to RH variations between 75% and 90%, and a sensitivity of ~10.15 nm/RH% could be obtained through linear fitting (with goodness of 0.961). This is nearly 10 times larger than the maximum sensitivity reported previously [26].

**Figure 5:**
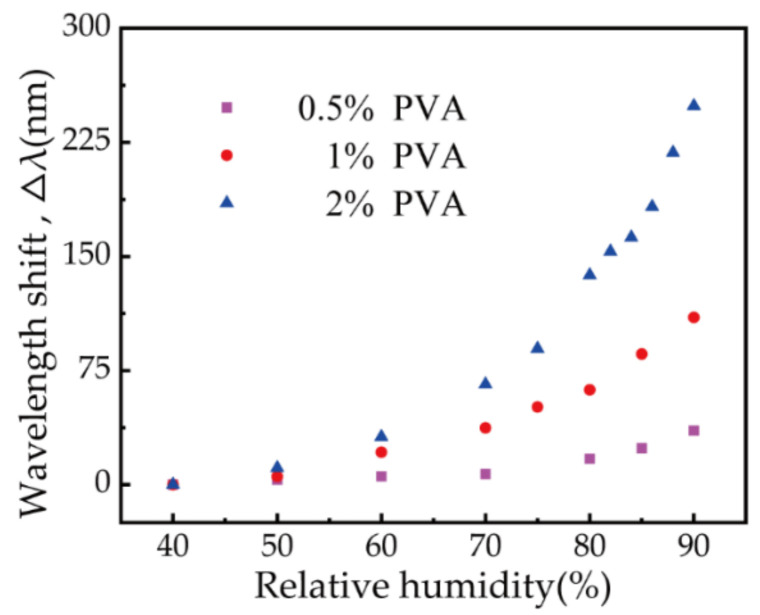
Resonant wavelength shift for samples 1 (0.5% PVA), 2 (1% PVA), and 3 (2% PVA) with increasing RH.

Each sample listed in Table 1 was placed in the chamber to test its RH response. Figure 5 shows the wavelength shift characteristics for the three samples as the RH increased from 40% to 90%. An initial resonant wavelength shift was evident in all three samples, and the increase in sensitivity was significant. In addition, the response of the device to humidity became increasingly sensitive as the thickness of the PVA film increased. The average sensitivity of each sample (RH ranging from 40% to 90%) was calculated to be 0.71 nm/RH%, 2.20 nm/RH%, and 4.98 nm/RH%, respectively. The sensor prepared with 2% (wt/wt) PVA solution exhibited the highest sensitivity of any sample. When the RH reached 90%, the resonant wavelength was close to the edge of the spectrometer’s working band. This indicated that if the thickness of the PVA film increases further, the resonant wavelength of the device may shift out of the range of the spectrometer at high humidity levels. The initial resonant wavelength of the fabricated device is briefly determined by the PVA film thickness. With a higher concentration of PVA solution and single dip coating, the obtained PVA film is thicker, while the resonant wavelength is longer, than that of the film fabricated with lower concentrations (also single dip coating). Generally, a fabricated device with a longer initial resonant wavelength exhibits higher sensitivity. In contrast, the PVA film thickness cannot be increased all the times, because the SPR will disappear when the PVA film thickness exceeds the evanescent field range. As such, the 2% (wt/wt) PVA sensor was selected for further evaluation.

**Figure 6:**
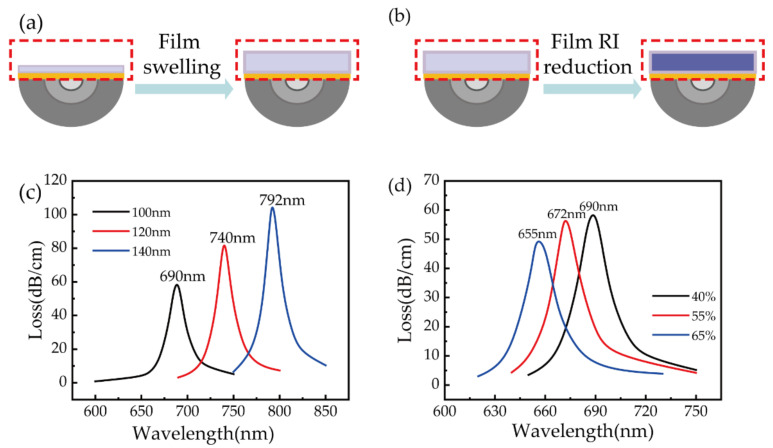
Schematic diagram of (**a**) PVA film swelling and (**b**) film RI reduction that cause a resonant wavelength shift of the proposed sensor. Simulated loss spectra of the RH sensor with (**c**) PVA film thickness set to 100, 120, and 140 nm and (**d**) RH of 40%, 55%, and 65%.

The reason for the sensitivity enhancement of our device is the low index of the polymer fiber compared to silica-fiber-based SPR sensors [29]. As mentioned above, two competitive mechanisms exist that can cause a SPR wavelength shift, film swelling and film RI reduction, as depicted in Figure 6a,b. On the one hand, due to the remarkable hygroscopic expansion characteristics of the PVA film, when PVA absorbs moisture, the film thickness increases, resulting in a redshift of the resonant wavelength. On the other hand, the moisture absorption of PVA leads to a decrease in the refractive index of the film, resulting in a blueshift of the resonant wavelength. To evaluate the contribution of these two mechanisms individually, simulations were conducted with a finite element method similar to that we reported earlier [33]. The simulation model was established based on the geometry of a polished POF with a residual thickness of 250 μm and a gold film thickness of 50 nm. The thickness of the PVA film in the model was set to be 100, 120, and 140 nm to calculate the SPR resonance wavelength to demonstrate the PVA-film-thickness-induced redshift of the SPR wavelength. Figure 6c shows the SPR wavelength shift considering film swelling only, and the results show the wavelength redshifted from 690 to 792 nm when the film thickness increased from 100 to 140 nm under a constant RH of 40%. This implies a wavelength redshift of about 25 nm with a film thickness swelling of 10%. Figure 6d shows the SPR wavelength shift caused by film RI reduction during RH measurement, where the PVA film thickness was set to 100 nm. When the RH increased from 40% to 65%, the RI of the PVA film decreased from 1.47 to 1.43 [31] and the SPR wavelength shifted from 690 to 655 nm. So, the wavelength shift purely induced by film RI reduction can be estimated to be about –1.4 nm for the RI change induced by 1%RH increment. According to the experimental and simulated results, it can be deduced that the sensor response to RH is dominated by film swelling during humidity measurement.

**Figure 7:**
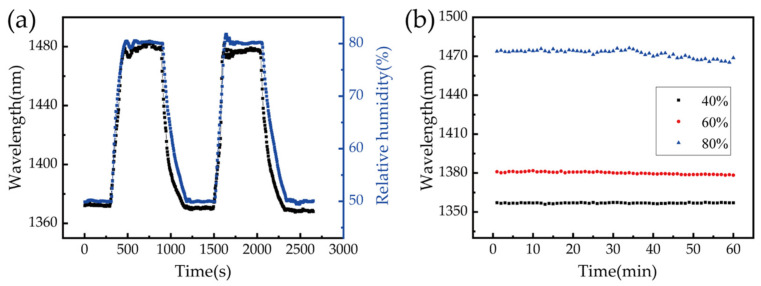
(**a**) The sensor stability test as the RH varied between 50% and 80%. The black curve shows the sensor response, and the blue curve shows data from the electric hygrometer. (**b**) Wavelength shifts in the resonance dip as the sensor was maintained at 40%, 60%, and 80% RH.

Stability is another important factor in the practical application of sensors. An experiment was designed to determine whether sensor performance would be affected by the shrinking and swelling of the PVA film. We also wanted to test the stability of the sensor over a long period. For the stability test, the sensor fabricated with 2% (wt/wt) PVA solution was placed in the homemade chamber and the RH was maintained at 50% for 5 min. The RH was increased to 80% and maintained for 5 min, then decreased to 50%. These steps were repeated multiple times as the transmitted spectrum, measured by the spectrometer, and RH values from the electric hygrometer were recorded by a PC every 5 s. Figure 7a shows the wavelength shift for the sensor and the real-time RH values corresponding to two cycles. The sensor wavelength shift was in excellent agreement with the data from the electric hygrometer, indicating the sensor exhibits good stability and no apparent degeneration over time. It can be seen clearly that the response of our fiber sensor is faster than that of the commercial hygrometer when the humidity drops. The stability of the proposed sensor was also tested by keeping the chamber at a specific RH level for extended periods. The RH in the chamber was maintained at 40%, 60%, and 80% for 1 h, with data recorded every 30 s. The stability of the sensor over this time period is shown in Figure 7b, where one can see clearly that the performance of the sensor was stable and showed only small fluctuations at an RH of 80%. This was primarily induced by the fluctuations of the RH environment in the chamber at high RH levels because it was difficult to maintain the RH in the chamber above an RH of 70% and the accuracy could only be controlled to within ±0.3% in our experiment.

**Figure 8:**
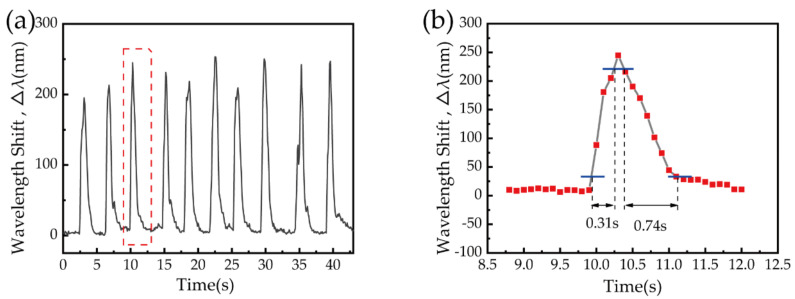
(**a**) Resonant wavelength shifts for the 2% (wt/wt) PVA sensor under several breaths. (**b**) The enlarged drawing of a single breathing process surrounded by the red frame in (**a**).

The applicability of our sensor to human breath sensing was also investigated. The sensor fabricated with 2% (wt/wt) PVA solution was fixed in a room environment by two fiber holders, at a temperature of 23.6 °C and an RH of 54%. The distance between the mouth and sensing area was kept to 20 cm, and periodic breathing was applied to the sensor, with the transmission spectrum recorded every 100 ms. Figure 8a shows the sensor wavelength shift for periodic breathing. Each peak in the figure represents a single breath, and the wavelength shift exceeded 200 nm, which is 10 times larger than that of a silica-fiber-based humidity sensor [34], indicating the sensor is extremely sensitive to human breathing. 

Additionally, the wavelength rapidly returned to its original position each time breathing ceased. Figure 8b shows an enlarged drawing of a single complete breathing process, surrounded by the red frame in Figure 8a. The wavelength shifted rapidly when breathing began and returned gradually to the RH of the room when breathing stopped. The device response time for this cycle of respiration was 0.31 s, and the time for recovery to the initial value (when breathing stops) was 0.74 s. To avoid randomness, 10 cycles of respiration, shown in Figure 8a, were statistically averaged, and the results show that the response time, recovery time, and the resonant wavelength shift were 0.44 ± 0.16 s, 0.86 ± 0.13 s, and 228.20 ± 4.61 nm, respectively. The response of the proposed sensor is faster than that of previously reported devices, as can be seen from Table 2.

**Figure 9:**
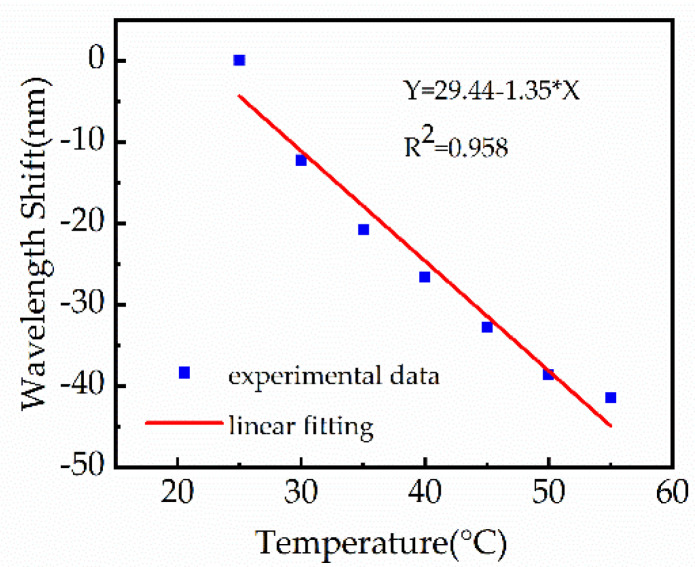
Sensor wavelength shifts as the temperature varied from 22 °C to 55 °C.

The 2% (wt/wt) PVA sensor was fixed in an oven as the surrounding temperature was increased from 22 °C to 55 °C in order to evaluate the cross-sensitivity caused by ambient temperature fluctuations. The temperature was increased by 5 °C in each interval and maintained for 5 min to ensure stability. Variations in the transmission spectrum of the sensor, in response to temperature changes, are shown in Figure 9. The resonance wavelength showed a clear blueshift as temperature increased. A linear fit was applied to the experimental data, with an R-squared value of 0.958. The temperature sensitivity of the sensor was measured to be 1.35 nm/°C, which corresponds to a cross-sensitivity of 0.13 RH%/°C for RH values of 75–90%. However, the air became drier as the temperature increased, which led to a decrease in the RH in the oven and induced a blueshift of the resonance wavelength. Thus, the actual temperature cross-sensitivity should be better than 0.13 RH%/°C. The temperature cross-sensitivity can be compensated possibly by introducing a fiber optic temperature sensor near the polished region of the POF, such as femtosecond-laser-inscribed fiber Bragg gratings [28].

Although the applied fabrication process did not contain unusual types of equipment, it was still hard to ensure reproducibility or even realize mass production at present for us, because the mechanical polishing and dip-coating techniques could hardly maintain the repeatability and stability of device fabrication. This implies that the price of the commercially available device fabricated this way will be higher. In contrast, the PVA layer was unstable and therefore the life time of the proposed structure will be short or it will be necessary to use special storage conditions, which will further increase the cost of the proposed device.

## 4. Conclusions

In conclusion, a PVA-coated RH sensor based on a polymer optical fiber was proposed and demonstrated. The device was highly sensitive to changes in RH between 40% and 90%, with an average sensitivity of 4.98 nm/RH%. The wavelength shift curve exhibited a linear response for RH values between 75% and 90%, where the sensitivity reached 10.15 nm/RH%. The performance of sensors prepared with varying PVA solution concentrations (0.5%, 1%, 2% (wt/wt)) was also tested. It was found that samples with thicker PVA films exhibit higher sensitivities. The stability of the PVA sensor was also reliable, and the cross-sensitivity caused by temperature fluctuations was less than 0.13 RH%/°C at 75–90% RH. The response to human breathing was also tested, and the device showed a wavelength shift of 228 ± 4.61 nm, with response and recovery times of 0.44 ± 0.16 s and 0.86 ± 0.13 s, respectively. In comparison with other optical fiber RH sensors, our device achieved high sensitivity, good repeatability, high stability, and low cross-sensitivity to temperature. In addition, as it was fabricated using a polymer optical fiber, its mechanical properties were highly beneficial. The device exhibits significant potential for practical sensing applications, including intensive care and human health analysis.

## Figures and Tables

**Table 1:** Parameters of the three samples used for subsequent tests.

Sample	Concentration of PVA (%)	Resonant Wavelength (nm)
1	0.5	613
2	1	712
3	2	1380

**Table 2:** Performance comparison of previously reported fiber optics humidity sensors.

Sensing Materials	Measurement Range (%RH)	Response Times	Sensitivity	Ref.
PVA	40–90	0.251 s	1.01 nm/%RH	[33]
CeO_2_/[Bmim]Br	11–97	4 s	0.455 nm/%RH	[35]
PVA	30–90	~2 s	0.737 nW/% RH	[12]
CoCl_2_-doped gelatine	50–80	<1 min	-	[36]
Rhodamine-B-doped hydroxypropyl cellulose	0–95	~2 min	-	[37]
TiO_2_	0–80	-	0.5 nm/%RH	[38]
PVA	50–90	1 min	0.066 dB/%RH	[39]
Agarose gel	30–80	<1 min	0.13 dB/%RH	[17]
Hydroxyethyl cellulose/polyvinylidenefluoride	20–80	<5 s	-	[40]
PVA	40–90	0.44 s	4.98 nm/%RH	This work

## References

[1] Khijwania S.K., Srinivasan K.L., Singh J.P. (2005). An evanescent-wave optical fiber relative humidity sensor with enhanced sensitivity. Sens. Actuators B Chem..

[2] Jagtap S., Rane S., Arbuj S., Gosavi S. (2018). Optical fiber based humidity sensor using Ag decorated ZnO nanorods. Microelectron. Eng..

[3] Li T., Dong X.Y., Chan C.C., Ni K., Zhang S.Q., Shum P.P. (2013). Humidity Sensor With a PVA-Coated Photonic Crystal Fiber Interferometer. IEEE Sens. J..

[4] Sun H., Yang Z., Zhou L., Liu N., Gang T., Qiao X., Hu M. (2015). A relative humidity sensing probe based on etched thin-core fiber coated with polyvinyl alcohol. Opt. Commun..

[5] Ma Q.F., Tou Z.Q., Ni K., Lim Y.Y., Lin Y.F., Wang Y.R., Zhou M.H., Shi F.F., Niu L., Dong X.Y. (2018). Carbon-nanotube/Polyvinyl alcohol coated thin core fiber sensor for humidity measurement. Sens. Actuators B Chem..

[6] Wu S., Yan G., Lian Z., Chen X., Zhou B., He S. (2016). An open-cavity Fabry-Perot interferometer with PVA coating for simultaneous measurement of relative humidity and temperature. Sens. Actuators B Chem..

[7] Bian C., Hu M.L., Wang R.H., Gang T.T., Tong R.X., Zhang L.S., Guo T., Liu X.B., Qiao X.G. (2018). Optical fiber humidity sensor based on the direct respose of the polyimide film. Appl. Opt..

[8] Huang J., Wang B.W., Ni K. (2018). Improving the Sensitivity of Humidity Sensor Based on Mach-Zehnder Interferometer Coated with a Methylcellulose. Int. J. Opt..

[9] Alwis L., Bremer K., Sun T., Grattan K.T.V. (2013). Analysis of the Characteristics of PVA-Coated LPG-Based Sensors to Coating Thickness and Changes in the External Refractive Index. IEEE Sens. J..

[10] Dissanayake K.P.W., Wu W.P., Nguyen H., Sun T., Grattan K.T.V. (2018). Graphene-Oxide-Coated Long-Period Grating-Based Fiber Optic Sensor for Relative Humidity and External Refractive Index. J. Lightwave Technol..

[11] Alwis L., Sun T., Grattan K.T.V. (2013). Fibre optic long period grating-based humidity sensor probe using a Michelson interferometric arrangement. Sens. Actuators B Chem..

[12] Dong X., Li T., Liu Y., Li Y., Zhao C.L., Chan C.C. (2011). Polyvinyl alcohol-coated hybrid fiber grating for relative humidity sensing. J. Biomed. Opt..

[13] Yan G., Liang Y., Lee E.H., He S. (2015). Novel Knob-integrated fiber Bragg grating sensor with polyvinyl alcohol coating for simultaneous relative humidity and temperature measurement. Opt. Express.

[14] Li T., Dong X.Y., Chan C.C., Zhao C.L., Zu P. (2012). Humidity Sensor Based on a Multimode-Fiber Taper Coated with Polyvinyl Alcohol Interacting with a Fiber Bragg Grating. IEEE Sens. J..

[15] Guan H., Xia K., Chen C., Luo Y., Tang J., Lu H., Yu J., Zhang J., Zhong Y., Chen Z. (2017). Tungsten disulfide wrapped on micro fiber for enhanced humidity sensing. Opt. Mater. Express.

[16] Wang X., Zhao C.L., Li J., Jin Y., Ye M., Jin S. (2013). Multiplexing of PVA-coated multimode-fiber taper humidity sensors. Opt. Commun..

[17] Bariain C., Matias I.R., Arregui F.J., Lopez-Amo M. (2000). Optical fiber humidity sensor based on a tapered fiber coated with agarose gel. Sens. Actuators B Chem..

[18] Shin J.C., Yoon M.S., Han Y.G. (2016). Relative Humidity Sensor Based on an Optical Microfiber Knot Resonator with a Polyvinyl Alcohol Overlay. J. Lightwave Technol..

[19] Zamarreno C.R., Hernaez M., del Villar I., Matias I.R., Arregui F.J. (2010). Tunable humidity sensor based on ITO-coated optical fiber. Sens. Actuators B Chem..

[20] Leal A., Theodosiou A., Frizera-Neto A., Pontes M.J., Shafir E., Palchik O., Tal N., Zilberman S., Berkovic G., Antunes P. (2018). Characterization of a new polymer optical fiber with enhanced sensing capabilities using a Bragg grating. Opt. Lett..

[21] Woyessa G., Fasano A., Markos C., Rasmussen H.K., Bang O. (2017). Low loss polycarbonate polymer optical fiber for high temperature FBG humidity sensing. IEEE Photonics Technol. Lett..

[22] Zhang W., Webb D.J. (2014). Humidity responsivity of poly (methyl methacrylate)-based optical fiber Bragg grating sensors. Opt. Lett..

[23] Theodosiou A., Komodromos M., Kalli K. (2018). Carbon cantilever beam health inspection using a polymer fiber Bragg grating array. J. Lightwave Technol..

[24] Miao Y.P., Liu B., Zhang H., Li Y., Zhou H.B., Sun H., Zhang W.H., Zhao Q.D. (2009). Relative Humidity Sensor Based on Tilted Fiber Bragg Grating with Polyvinyl Alcohol Coating. IEEE Photonics Technol. Lett..

[25] Wong W.C., Chan C.C., Chen L.H., Li T., Lee K.X., Leong K.C. (2012). Polyvinyl alcohol coated photonic crystal optical fiber sensor for humidity measurement. Sens. Actuators B Chem..

[26] Tang L., Feng Y., Xing Z., Chen Z., Yu J., Guan H., Lu H., Fang J., Zhong Y. (2018). High-sensitivity humidity sensing of side-polished optical fiber with polymer nanostructure cladding. Appl. Opt..

[27] Gravina R., Testa G., Bernini R. (2009). Perfluorinated Plastic Optical Fiber Tapers for Evanescent Wave Sensing. Sensors.

[28] Nan Y., Kinet D., Chah K., Chapalo I., Caucheteur C., Mégret P. (2021). Ultra-fast fiber Bragg grating inscription in CYTOP polymer optical fibers using phase mask and 400 nm femtosecond laser. Opt. Express.

[29] Cao S., Shao Y., Wang Y., Wu T., Zhang L., Huang Y., Zhang F., Liao C., He J., Wang Y. (2018). Highly sensitive surface plasmon resonance biosensor based on a low-index polymer optical fiber. Opt. Express.

[30] Jesswein I., Hirth T., Schiestel T. (2017). Continuous dip coating of PVDF hollow fiber membranes with PVA for humidification. J. Membr. Sci..

[31] Zhao C., Yuan Q., Fang L., Gan X., Zhao J. (2016). High-performance humidity sensor based on a polyvinyl alcohol-coated photonic crystal cavity. Opt. Lett..

[32] Alwis L., Sun T., Grattan K.T.V. (2013). Design and performance evaluation of polyvinyl alcohol/polyimide coated optical fibre grating-based humidity sensors. Rev. Sci. Instrum..

[33] Shao Y., Wang Y., Cao S., Huang Y., Zhang L., Zhang F., Liao C., Wang Y. (2018). Mechanism and Characteristics of Humidity Sensing with Polyvinyl Alcohol-Coated Fiber Surface Plasmon Resonance Sensor. Sensors.

[34] Rivero P.J., Urrutia A., Goicoechea J., Matias I.R., Arregui F.J. (2013). A Lossy Mode Resonance optical sensor using silver nanoparticles-loaded films for monitoring human breathing. Sens. Actuators B Chem..

[35] Xie W. (2017). CeO2/ionic liquid hybrid materials with enhanced humidity performance. Sens. Actuators B Chem..

[36] Russell A.P., Fletcher K.S. (1985). Optical sensor for the determination of moisture. Anal. Chim. Acta.

[37] Otsuki S., Adachi K., Taguchi T. (1998). A novel fiber-optic gas-sensing configuration using extremely curved optical fibers and an attempt for optical humidity detection. Sens. Actuators B Chem..

[38] Alvarez-Herrero A., Guerrero H., Levy D. (2004). High-sensitivity sensor of low relative humidity based on overlay on side-polished fibers. IEEE Sens. J..

[39] Gastón A., Pérez F., Sevilla J. (2004). Optical fiber relative-humidity sensor with polyvinyl alcohol film. Appl. Opt..

[40] Muto S. (2003). A plastic optical fibre sensor for real-time humidity monitoring. Meas. Sci. Technol..

